# Measuring the change in deleterious mutation burden of barley germplasm conserved over one regeneration cycle

**DOI:** 10.1093/g3journal/jkag151

**Published:** 2026-06-15

**Authors:** Yong-Bi Fu

**Affiliations:** Plant Gene Resources of Canada, Saskatoon Research and Development Centre, Agriculture and Agri-Food Canada, Saskatoon, SK, Canada S7N 0X2

**Keywords:** deleterious mutation, mutation burden, mutation accumulation, plant germplasm conservation, germplasm regeneration

## Abstract

Deleterious mutations will occur and accumulate in germplasm conserved under long-term cold storage. However, little is known about the extent of deleterious mutation accumulation in conserved germplasm. This study represents an attempt to measure the change in deleterious mutation burden of barley germplasm conserved over 1 regeneration cycle. Specifically, seed-based RNA-Seq analysis was performed on paired samples of 90 barley accessions representing germplasm conserved under cold storage since its original acquisition (G0) and since the first regeneration (G1). The analysis identified 1,642 deleterious variants by 2 SNP calling methods. These deleterious variants were widely distributed across 7 chromosomes, were present in only 8 or fewer samples, occurred predominantly in heterozygotes, were associated with diverse biological processes, and were largely predicted to be weakly detrimental. Based on BCFtools-based SNP calls, the change in total mutation burden per deleterious locus per conservation year was estimated at 0.00123, with a standard deviation of 0.00084, for an accession conserved through the first regeneration. This estimate suggests that a conserved barley accession accumulated 28 to 37 deleterious variants over the G0 to G1 period. More deleterious variants were found in G1 than in G0 samples; variants unique to each sample type were also observed; and a high proportion of variants occurred in heterozygotes. These findings clearly demonstrated the technical and practical feasibility of measuring mutation accumulation in barley germplasm conserved in a genebank.

## Introduction

Deleterious mutations will occur in more than 7 million plant germplasm accessions conserved in 2,000 genebanks worldwide (FAO 2010; Engels and Ebert 2024), particularly in those conserved under long-term cold storage, as a result of changes in DNA that disrupt normal gene function (Ashton 1956; Roberts 1978; Dourado and Roberts 1984). These mutations can accumulate in conserved germplasm from the joint actions of selection and genetic drift, both before and during germplasm regeneration (Schoen et al. 1998). Mutation accumulation will alter the original genetic background of conserved germplasm (Charlesworth et al. 1993), potentially increasing its vulnerability to future environmental pressures by reducing reproductive success and survival (Hoffman et al. 2004; Lu et al. 2006; Roles and Conner 2008; Moyers et al. 2018; Kono et al. 2019; Dwivedi et al. 2023). This accumulative genetic change could compromise some objectives of the long-term germplasm conservation mission of minimizing genetic shift and reducing loss of the original genotypes (Schoen and Brown 2001; Fu 2024a). Therefore, there is a clear need to develop effective genebank management procedures to minimize the genetic changes from deleterious mutation accumulation (Fu 2024a). However, the knowledge of deleterious mutation accumulation in conserved germplasm is lacking (Fu et al. 2023; Fu and Horbach 2025).

Assessing mutation burden (or the extent of deleterious mutations) was traditionally quite challenging (Mukai 1964; Charlesworth et al. 1990; Kondrashov and Kondrashov 2010) but has become both technically possible and practically feasible. This progress reflects advances in genomics, particularly through genetic load studies in the human genome (eg Cooper et al. 2005; Henn et al. 2015), as well as the development of bioinformatics tools for identifying deleterious genetic variants (eg Ng and Henikoff 2003). Technically, genome-wide genetic variants are screened across a sequenced genome and then identified to be deleterious through gene function prediction of a nonsynonymous site change alone (eg Ng and Henikoff 2003) and/or in combination with the intensity of purifying selection inferred from phylogenetic restraints on the site (Davydov et al. 2010). Over the last decade, numerous studies have been conducted to identify genome-wide deleterious genetic variants in many plant genomes (Mezmouk and Ross-Ibarra 2014; Renaut and Rieseberg 2015; Kono et al. 2016; Liu et al. 2017; Ramu et al. 2017; Valluru et al. 2019; Lozano et al. 2021; Fu et al. 2023; Sun et al. 2023; Wu et al. 2023; Razifard et al. 2025). These predicted deleterious mutations are more likely to be harmful (Kono et al. 2019; Plekhanova et al. 2019; Sun et al. 2023), although their fitness effects are largely unknown (Roles and Conner 2008; Bertorelle et al. 2022). Overall, these studies have successfully demonstrated the effectiveness of identifying deleterious genetic variants across plant genomes and have provided informative estimates of mutation burden in plant germplasm (eg Ramu et al. 2017; Kistler et al. 2018; Sun et al. 2023; Wu et al. 2023).

In genebanks, plant germplasm is typically conserved in the form of seeds and managed following Food and Agriculture Organization (FAO) Genebank Standards (FAO 2014, 2022). When seed of an accession is acquired for conservation, it must go through various genebank procedures, including seed cleaning and increase, before being packaged in a sealed envelope at an assumed 20% relative humidity and stored at −18 °C to −20 °C for long-term conservation. Seed viability of conserved germplasm is monitored over time (Hay and Whitehouse 2017). When the seed viability is below the threshold value (commonly 85%), part of the seed sample for the accession will be regenerated in the greenhouse or field. The newly regenerated seed will be packaged as a new sample for the originally acquired accession for long-term cold storage. Regeneration and storage procedures are repeated over time to maintain germplasm viability and availability for future use. This process provides a unique opportunity to assess the changes in the mutation burden of conserved germplasm over regeneration cycles, as seed samples from multiple regenerations of the same accession commonly exist in genebanks.

To enhance the long-term management of plant germplasm conserved in worldwide genebanks, an attempt was made to measure the change in deleterious mutation burden of barley (*Hordeum vulgare* L.) germplasm conserved over 1 regeneration cycle using seed-based RNA-Seq analysis (Wang et al. 2009). Specifically, 90 barley accessions with paired samples (1 conserved since the original acquisition and 1 since the first regeneration cycle) were selected from the barley collection conserved at Plant Gene Resources of Canada (PGRC), Saskatoon, Canada. RNA-Seq analysis was applied to 180 barley samples, and genome-wide deleterious variants were identified based on phylogenetic restraints. Total mutation burden per deleterious locus was estimated for each assayed sample. The pairwise sample differences in total mutation burden per accession were calculated using a novel method that adjusts for variable years of conservation to estimate the change occurring over 1 germplasm regeneration. Overall, this seed-based RNA-Seq analysis was undertaken with the hope to assess the technical feasibility of measuring the extent of deleterious mutation accumulation in plant germplasm conserved in a genebank.

## Materials and methods

### Sample selection and acquisition

We selected 90 barley accessions (Table 1) from the PGRC barley collection based primarily on 2 factors: the number of regeneration cycles and the availability of seed for distribution. These accessions were originally acquired by PGRC from 8 countries between 1973 and 1989, conserved in sealed envelopes at an assumed 20% relative humidity under PGRC long-term storage at −18 °C and subjected to only one regeneration cycle between 1990 and 2016, following FAO Genebank Standards (FAO 2014). Specifically, when an original accession was identified with seed viability of 85% or lower, approximately 200 seeds were randomly sampled from the accession and regenerated at the experimental farm of the Saskatoon Research and Development Centre with a field layout of 1 single 3-m row plot per accession with 60 cm between rows, along with strict control of weeds, pests, and diseases. Seeds were harvested from all plants in the row for each accession. Planting 90 to 210 seeds is expected to retain alleles with frequencies between 0.003 and 0.05 across 10 to 150 loci with a probability of 90% to 95% (Crossa et al. 1993). This regeneration procedure was repeated over the years because the selected accessions were not regenerated in the same year.

**Table 1.** List of 90 barley accessions assayed in 2021 with their paired samples representing the conserved germplasm since its original acquisition (G0; from 1973 to 1989) and since the first regeneration cycle (G1; from 1990 to 2016), along with their countries of origin and accession descriptions.

Accession	Origin	Description	Year0	Year1	Accession	Origin	Description	Year0	Year1
CN9448	Canada	Beacon	1980	1994	CN31558	United States	Betzes	1978	1995
CN9450	Canada	Betzes	1980	1993	CN31673	United States	Atlas 46	1978	1994
CN9456	Canada	B.T.322	1980	1993	CN32160	Germany	Nackta	1978	1995
CN9479	Canada	Olli	1980	1993	CN32585	Russia	Winer	1978	1995
CN9487	Canada	Vantmore	1980	1993	CN33302	Canada	U48/54	1973	1995
CN12930	Canada	C18-73	1983	2009	CN33306	Canada	Fjola	1973	1995
CN13176	Canada	C269-22	1983	1990	CN35778	Azerbaijan	Pallidum 596	1979	2003
CN13180	Canada	C273-3	1983	1990	CN35779	Russia	Toomas	1979	1994
CN13203	Canada	D12-3	1983	1990	CN35780	Russia	Zavet	1979	1994
CN13224	Canada	D29-2	1983	1990	CN39286	Canada	Bichita	1982	1996
CN13372	Canada	F93-8	1983	1990	CN39309	Canada	QB179.97	1982	1994
CN14132	Canada	S55-G90	1983	2016	CN39314	Canada	QB722.7	1982	1994
CN14393	Canada	S99-G184	1983	1999	CN39339	Germany	Volla	1982	1994
CN14494	Canada	S112-G364	1983	1999	CN39379	Canada	BT911	1982	1994
CN14508	Canada	S114-G20	1983	1999	CN39408	Canada	M7619-4337	1982	1994
CN14527	Canada	S114-G122	1983	1999	CN39410	Canada	SB80211	1982	1994
CN14648	Canada	S144-4	1983	1999	CN39413	Canada	SF79201	1982	1995
CN14653	Canada	S148-48	1983	1999	CN40276	Russia	PGR 13302	1983	1994
CN14690	Canada	S193-1	1983	2011	CN40278	Russia	WIR 4514	1983	1994
CN14913	Canada	S309-G76	1983	2011	CN40300	United States	PGR 13334	1983	1994
CN15040	Canada	S379-17	1983	2014	CN42483	Denmark	Binder	1984	1994
CN15059	Canada	S382-7	1983	2011	CN42525	United States	Composite Cross	1984	1994
CN15073	Canada	S383-15	1983	2014	CN42855	Canada	SM82061	1984	1994
CN15140	Canada	S535-59	1983	2011	CN42877	United States	Advance	1984	1994
CN16296	Canada	W248-78	1983	2013	CN42934	Canada	Jackson	1985	1994
CN16530	Canada	B17-6	1984	2013	CN43235	Canada	SM83091	1985	1994
CN16547	Canada	B25-41	1984	2013	CN43288	Canada	TBM80-19	1985	1994
CN16562	Canada	C21-21	1984	2013	CN43556	Australia	Ilineckii 43	1986	1994
CN16563	Canada	C45-1	1984	2013	CN43634	Canada	82-RCBB-13	1986	1994
CN16700	Canada	C398-13	1984	2016	CN43666	Canada	OB752-21	1986	1994
CN16873	Canada	D259-5	1984	2016	CN43731	Canada	SM84275	1986	1994
CN17537	Canada	S709-51	1984	2014	CN43743	Canada	H69072193	1986	1994
CN17557	Canada	S779-41	1984	2014	CN43747	United States	2B81-4038	1986	1994
CN17580	Canada	S783-22	1984	2014	CN45881	Canada	TR 115	1988	1994
CN17600	Canada	S791-3	1984	2014	CN45883	Canada	C.D.C. Guardian	1988	1994
CN17616	Canada	S899-1	1984	2014	CN45886	Canada	TR 227	1988	1994
CN17628	Canada	S925-45	1984	2014	CN45889	Canada	TR 311	1988	1994
CN17630	Canada	S933-9	1984	2014	CN45892	Canada	92-41	1988	1994
CN17638	Canada	S973-5	1984	2014	CN46226	Canada	Clark	1989	1994
CN17820	Canada	L06/L64-1	1985	1994	CN46747	Canada	OB959-7	1989	1994
CN17822	Canada	BT 364	1985	1994	CN46748	Canada	OB956-13	1989	1994
CN31045	Canada	PGR 2057	1976	1994	CN46750	Canada	OB758-17	1989	1994
CN31054	Canada	Saxonia	1976	1994	CN46753	Canada	OB762-13	1989	1994
CN31058	Canada	12502 C/2	1976	1994	CN51811	Canada	Brier	1989	1994
CN31140	France	Sandra	1977	1994	CN52167	France	Patty	1989	1994

Year0 and Year1 show the years of the original accession acquisition and the first completed regeneration cycle. For each accession, *Y*_0_ = 2021−Year0; *Y*_1_ = 2021−Year1; and *Y*_R_ = *Y*_0_−*Y*_1_.

Seed for these 90 accessions, with paired samples representing germplasm conserved from the time of original acquisition (G0) and from the first regeneration cycle (G1), along with their inventory data (Table 1), such as passport information, country of origin, year of acquisition (Year0), and year of the first regeneration (Year1), was acquired in October 2021 from Mr Brian James at PGRC, following the Standard Material Transfer Agreement of the International Treaty on Plant Genetic Resources for Food and Agriculture (https://www.fao.org/plant-treaty/areas-of-work/the-multilateral-system/smta/en/; accessed 15 May 2026).

### RNA-Seq analysis

From each of the 180 barley samples, 10 seeds were randomly selected, crushed between pieces of copy paper with a hammer, and ground into a fine powder using a mortar and pestle with liquid nitrogen. Approximately 50 mg of ground tissue from each sample was transferred into a 2-mL tube containing grinding beads (RNeasy PowerPlant Kit, QIAGEN Inc., Toronto, ON, Canada). Samples were flash-frozen in liquid nitrogen and stored at −80 °C until RNA extraction. Total RNA was extracted using the RNeasy PowerPlant Kit with the following modifications: (i) ground tissue was further disrupted using a TissueLyser II (QIAGEN Inc.) at 20 Hz for 5 min and (ii) RNA was eluted twice with 50 µL RNase-free water and combined prior to quantification. RNA concentration was assessed with a Thermo Scientific NanoDrop 8000 (Thermo Fisher Scientific, Mississauga, ON, Canada) and purity was assessed using the 260/280 and 260/230 absorbance ratios. RNA integrity number was determined using the RNA 6000 Nano Kit on an Agilent 2100 Bioanalyzer (Agilent Technologies, Mississauga, ON, Canada). RNA samples were stored at −80 °C until library preparation.

mRNA-Seq libraries were prepared using the SENSE mRNA-Seq Library Prep Kit V2 (Lexogen, Greenland, NH, USA) with 12-nt Unique Dual Index sets A1, A2, and A3, following the manufacturer's protocol. cDNA amplification was performed with 19 to 20 PCR cycles for samples containing ≥1,000 ng of total RNA, and 23 cycles for those <1,000 ng. Final libraries were quantified using the Invitrogen Quant-iT PicoGreen dsDNA assay kit (Thermo Fisher Scientific) and then pooled into a multiplex library and assessed using an Agilent 2100 Bioanalyzer with a High-Sensitivity DNA chip. To improve the library quality, the multiplex library was further cleaned to remove small fragments (<250 bp) using 2 rounds of KAPA Pure Beads (Roche Canada, Mississauga, ON, Canada) at a 0.9× beads-to-sample ratio, following the single-tail clean-up protocol. Sequencing was performed at the Centre d’expertise de la services Génome Québec on a single lane of an Illumina NovaSeq 6000 S4 flow cell, generating 100 bp paired-end reads. Raw sequence data was deposited in June 2025 to the National Center for Biotechnology Information Sequence Read Archive under the BioProject ID PRJNA1274897.

### SNP calling

RNA sequencing generated a pair of demultiplexed forward and reverse FASTQ files for each sample. FASTQ files were assessed with FastQC v0.11.9 (Andrews 2010) and summarized for each read direction using MultiQC v1.10.1 (Ewels et al. 2016). The FASTQ files were processed using Trimmomatic v0.32 (Bolger et al. 2014) to remove any adapter sequences, trim low-quality bases (below a Phred score of 24), and remove any sequences shorter than 60 bp. The following trim settings were used: ILLUMINACLIP: TruSeq3-PE-2.fa; SLIDINGWINDOW:10:24; and MINLEN:60. FastQC was rerun after trimming to verify the removal of the Illumina adapter sequences. A split-chromosome version of the barley reference (Mascher et al. 2017) was obtained from Dr Martin Mascher, IPK Gatersleben, Germany. The split-chromosomes have the same number of bases and are similarly masked as the “160404_barley_pseudomolecules_masked.fasta” (Mascher et al. 2017) and the EnsemblPlants v40 version of the reference. However, the split-chromosome reference was used to circumvent the 2^39^−1 base (≈536 Mb) limit of SAMtools (Li et al. 2009, https://www.htslib.org) binary alignment map (BAM) indexing. A graph Ferragina–Manzini index of the genome reference was generated using Hisat2-build (Kim et al. 2019), and alignments against the split genome sequences were performed using Hisat2 (Kim et al. 2019), a fast, splice-aware aligner. The resulting BAM files were filtered to remove PCR duplicates using the MarkDuplicates tool from the Genome Analysis Toolkit v4.2.6.1 (Van der Auwera et al. 2013). SAMtools sort was applied to produce sorted BAM files.

To increase the accuracy of identifying deleterious variants, SNP calling was performed using 2 commonly applied methods: BCFtools v1.9 (Danecek et al. 2021) and ANGSD v0.935 (Korneliussen et al. 2014). BCFtools is one of the most widely used variant calling and SNP genotyping tools, based on estimated genotype likelihoods at each genomic position, and is therefore expected to generate SNP data sets with missing data. ANGSD applies a probabilistic framework to call SNPs based on estimated genotype likelihoods by optimizing allele frequencies and commonly generates SNP data sets without missing data; however, mapping bias (favoring reference over alternative alleles) may occur (Günther et al. 2025). Specifically, BCFtools was used with the following command: bcftools mpileup -Ou -f -b | bcftools call -vmO z -V indels -o. To control SNP quality and the level of missing data, Vcftools v0.1.15 (Danecek et al. 2011) was applied with the following command: vcftools –vcf input.vcf –out output.vcf –recode-INFO-all –max-alleles 2 –min-alleles 2 –minDP 10 –minQ 20 -max-missing 80 –recode. This filtering generated a BCFtools-based data set of SNPs with up to 20% missing values. For the ANGSD application, the following parameters were selected: -dovcf 1 -gl 1 -dopost 1 -domaf 1 -doMajorMinor 4 -doGeno 3 -snp_pval 1e-6, generating an ANGSD-based SNP data set without missing values. These 2 methods of SNP calling were applied to all 180 barley sample BAM files based on the split-chromosome reference, and the generated variant call format (VCF) files were then realigned to full-length chromosomes with a custom Perl script.

### Identification of deleterious SNPs

For both SNP data sets, annotation was performed using the stand-alone Ensembl Variant Effect Predictor (VEP) (McLaren et al. 2016; Naithani et al. 2017) from the generated SNP VCF file. The VEP output also included the score of the sorting intolerant from tolerant (SIFT) algorithm (Vaser et al. 2015) for each annotated SNP. The SIFT score measures the predictive impact of an amino acid substitution and can distinguish between functionally neutral and deleterious amino acid changes. An amino acid substitution with a SIFT score of ≤0.05 is predicted to be deleterious. This study also applied a combination of SIFT scores and GERP++ Rejected Substitution (RS) scores (Davydov et al. 2010) to identify deleterious SNPs (dSNPs). The RS scores for the extremely conserved chromosomal regions of the barley genome were previously generated using GERP++ based on reference genomes of 12 plant species to measure the phylogenetic conservation from the substitution of a locus (Fu et al. 2023). These published RS scores provide a quantification of the conservation of each nucleotide in a multispecies alignment. A positive score (RS >0) at a substitution site means fewer substitutions than expected. Thus, substitutions occurring at a conserved site with RS >0 are predicted to be deleterious, with higher RS scores indicating increasingly deleterious effects. Combining SIFT (<0.05) and GERP++ RS (>0) annotations (or SIFT-RS combination) enabled more accurate identification of dSNPs in constrained portions of the genome. In this study, an additional step was included to identify multiple data sets of dSNPs that were shared between the BCFtools-based and ANGSD-based SNP data sets, depending on variable levels of missing SNP data. The shared dSNPs are not only expected to have an improved accuracy in the identification of deleterious variants, but also can provide more informative comparisons of mutation burden estimations based on different SNP genotyping methods.

### Characterizing the identified dSNPs

For each SNP genotype VCF file, the total detected SNPs were counted for each chromosome and plotted using R hist() (R Core Team 2024). Similarly, for each deleterious SNP genotype VCF file, the shared dSNPs were also counted for each chromosome. The SNP counting was done with a custom shell script. For BCFtools-based dSNP data sets, minor allelic frequencies of dSNPs in the assayed samples were analyzed using Vcftools and plotted using R plot(). Similarly, minor allelic frequencies of ANGSD-based shared dSNPs were also analyzed to compare their frequency distributions. To understand the detrimental severity of the shared dSNPs, their RS values were also extracted and plotted using R plot() to assess their frequency distribution.

Gene ontology (GO) enrichment analysis was also performed on the dSNPs that were shared between BCFtools- and ANGSD-based SNP data sets. The first step was to identify and extract genes associated with the shared dSNPs from the barley gene annotation file using a custom shell script. The associated gene list was analyzed using the online tool agriGO v2.0 (Tian et al. 2017) to generate enriched GO terms. The resulting significant GO term sets were further analyzed and visualized using the online tool REVIGO (Supek et al. 2011) with treemaps to assist in the interpretation of gene enrichments and functions.

### Method for estimating mutation burden change

A new method was specifically developed to estimate mutation burden change over 1 regeneration cycle using paired G0 and G1 samples with varying durations of cold storage conservation. G0 and G1 samples could differ in the number of conservation years since original acquisition (*Y*_0_) and since the first germplasm regeneration (*Y*_1_), respectively. Consequently, the number of conservation years between acquisition and the first regeneration, *Y*_R_ (=*Y*_0_−*Y*_1_), also varies among the assayed accessions. These differences in *Y*_0_, *Y*_1_, and *Y*_R_ reflect the variations in the timing of accession acquisition, regeneration, and storage. For example, 2 accessions, A and B, were acquired 40 and 30 yr ago, respectively, and underwent their first regenerations 15 and 20 yr ago, resulting in differing conservation year values (*Y*_0A_ = 40 vs *Y*_0B_ = 30; *Y*_1A_ = 15 vs *Y*_1B_ = 20; and *Y*_RA_ = 25 vs *Y*_RB_ = 10).

Following the commonly used measure of mutation burden (eg Ramu et al. 2017), the total mutation burden per deleterious locus $M_{ti}$ for a sample representing accession *i* is estimated as the proportion of the predicted deleterious variants over all the identified variants in the sample as:


(1) $$M_{ti}=\frac{C_{\mathrm{het},i}+2C_{\mathrm{hom},i}}{2L}$$

where $C_{\mathrm{het},i}\;\mathrm{and}\;C_{\mathrm{hom},i}$ are the counts of deleterious variants in heterozygous and homozygous loci in the G0 or G1 sample of the accession *i* (*i* = 1 to *n*) for all diallelic deleterious loci *L*. The change in total mutation burden per deleterious locus per conservation year between the paired G0 and G1 samples of 1 accession *i* is measured as $DM_{tyi}$:


(2) $$DM_{tyi}=\frac{M_{t1i}}{Y_{1i}}-\frac{M_{t0i}}{Y_{0i}}$$

Note that $M_{t1i}$ and $M_{t0i}$ are the total mutation burdens per deleterious locus for the G1 and G0 sample of the accession *i*, respectively. As $DM_{tyi}$ may be correlated with the years of conservation from G0 to G1 ($Y_{Ri}$) for the accession, the change should be adjusted for $Y_{Ri}$. Thus, the $DM_{tyi}$ that adjusts for variable years of conservation from G0 to G1 per accession is redefined as:


(3) $$DM_{tyRi}=DM_{tyi}*\left(\frac{\bar{Y_{R}}}{Y_{Ri}}\right)$$

Note that $\bar{Y_{R}}=\frac{1}{n}\sum_{i}^{n}Y_{Ri}$, the mean of variable conservation years $Y_{Ri}$ for all accessions. The average change across all assayed accessions with variable years of conservation (*Y*_0_, *Y*_1_, and *Y*_R_) over 1 regeneration cycle is calculated as:


(4) $$DM_{tyR}=\frac{1}{n}\overset{n}{\underset{i}{\sum }}\;DM_{tyRi}$$

$DM_{tyRi}$ from Equation (3) is scaled to a deleterious locus and a conservation year and has the unique property of being independent of the number of deleterious mutations *L* being identified and the number of years under conservation (*Y*_0_, *Y*_1_, and *Y*_R_) for each sample. Thus, $DM_{tyR}$ from Equation (4) not only allows for an informative comparison of the mutation burden changes estimated from different studies but also makes the projection of total mutation burden in conserved germplasm feasible with the assumption of variable levels of deleterious mutations and different conservation scenarios. A positive or negative $DM_{tyR}$ value indicates the accumulation or disappearance of deleterious alleles in a conserved accession after regeneration, respectively.

For this study, the 3 conservation years (*Y*_0_, *Y*_1_, and *Y*_R_) for each accession were calculated from Table 1. Counting deleterious variants in heterozygotes and homozygotes across all deleterious loci for each sample was performed using a custom shell script from each dSNP data set, so $M_{ti}$ was obtained using Equation (1) for each G0 or G1 sample of the accession *i*. For BCFtools-based dSNP data sets, $M_{ti}$ was calculated based only on the effective deleterious loci without missing SNP data. $DM_{tyi}$ and $DM_{tyRi}$ were estimated using Equations (2 and 3), respectively, for the accession *i* from each dSNP data set. The average change $DM_{tyR}$ was estimated using Equation (4) across all 90 accessions. To validate the need for adjusting for *Y*_R_ for $DM_{tyR}$, a linear regression analysis of $DM_{tyi}\;$ over $Y_{Ri}$ was made in each dSNP data set to determine the significance and strength of their correlation. The acquired estimates of $\;DM_{tyRi}$ were further analyzed for their distributions in each dSNP data set using R barplot() and tested for (i) their correlations between BCFtools-based and ANGSD-based dSNP data sets using R lm(), (ii) significance of their median larger than zero using Wilcoxon signed-rank test implemented with R wilcox.test(), (iii) their differences between 2 dSNP data sets using Wilcoxon signed-rank test implemented with Wilcoxsign_test() in R package coin, and (iv) their overall differences among different dSNP data sets using nonparametric Kruskal–Wallis test with R kruskal.test(). Comparative analyses of $DM_{tyR}$ estimates were also made with respect to the extent of missing data and SNP calling method.

### Assessing allelic changes from G0 to G1 samples

To understand the changes of deleterious alleles over 1 regeneration cycle, we conducted several comparative counts for each dSNP data set. Specifically, we quantified (i) the numbers of deleterious loci and alleles in G0, G1, and G0+G1 samples; (ii) deleterious alleles and loci shared between G0 and G1; (iii) deleterious alleles detected in G1 but undetected in G0 and their associated loci; and (iv) deleterious alleles detected in G0 but undetected in G1 and their associated loci. We also counted deleterious alleles in homozygotes and heterozygotes and estimated the contributions of each to total mutation burden without considering the conservation years. As only one single G0–G1 sample pair for a heterogenous accession was assayed and the paired G0–G1 sample did not originate from the same initial plant, these comparative counts are not informative for determining whether an observed allelic turnover resulted from *de novo* mutation, standing variation, or a sampling effect. However, these analyses enable an assessment of the extent of allelic maintenance and turnover from G0 to G1, thus enhancing our understanding of mutation dynamics in conserved germplasm.

## Results

### SNP identification and annotation

**Fig. 1.**
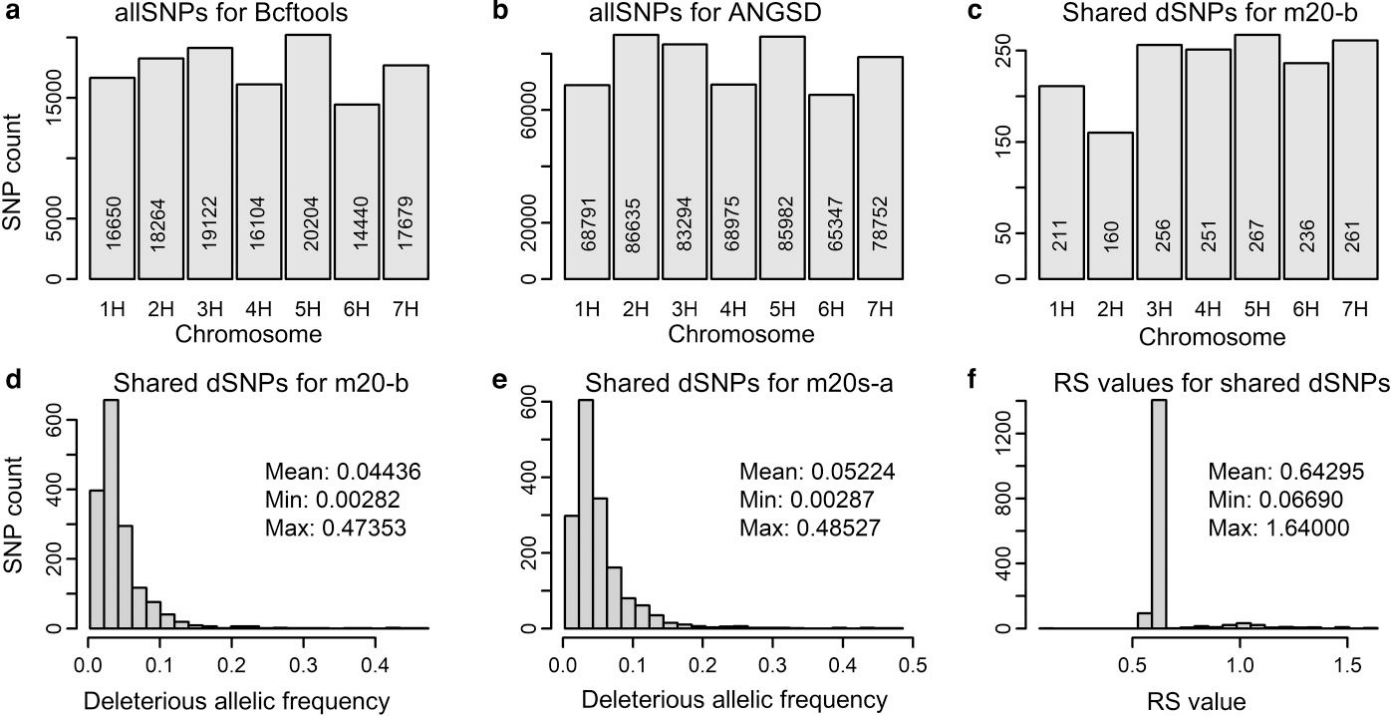
The chromosomal distributions of all identified SNPs from BCFtools with up to 20% missing data (a) and from ANGSD (b) and predicted dSNPs (c), frequency distributions of shared dSNPs (d and e), and frequency distributions of GERP++ RS values for the shared dSNPs (f). Each panel is labeled with its specific data set. a and b) Show the chromosomal distributions of all SNPs from BCFtools and ANGSD, respectively. c) Illustrates the chromosomal distributions of dSNPs shared between BCFtools- and ANGSD-based data sets. d and e) Show the frequency distribution of shared dSNPs in m20-b and m20s-a data sets, respectively.

RNA-Seq produced 2.29 billion sequence reads for the 180 barley samples with an average of 12.7 million sequence reads per sample (Supplementary Table 1). Specifically, there were an average of 8.4 and 9.0 million mapped sequence reads for each G0 and G1 sample, respectively. SNP calling by BCFtools identified 513,997 SNPs with up to 99.4% missing data across the 180 samples. After filtering based on SNP quality and missing data, there were 122,463, 72,431, 42,763, and 4,768 SNPs with up to 20%, 10%, 5%, and 0% missing data, respectively. In contrast, SNP calling by ANGSD revealed 537,776 SNPs without missing data. The identified SNPs were widely distributed across all 7 barley chromosomes in both BCFtools- and ANGSD-based SNP data sets and displayed similar chromosomal distributions, as illustrated in Fig. 1a and b.

VEP-based annotation analyses of the identified SNPs were performed on BCFtools-based data sets of SNPs with up to 20% missing data and ANGSD-based SNP data set. These VEP analyses allowed for the classification of SNPs into up to 17 different classes with the most severe consequences (Table 2). The amount of SNPs per class varied substantially among the 17 classes and 2 SNP data sets representing the 2 SNP calling methods. For example, there was a higher proportion of missense_variant over total SNPs identified in the BCFtools-based SNP data set (0.302) than in the ANGSD-based SNP data set (0.257). Similarly, there was a lower proportion of loss-of-function variants over total SNPs identified in the BCFtools-based SNP data set (0.027) than in the ANGSD-based SNP data set (0.040).

**Table 2.** Annotations of SNPs generated by BCFtools and ANGSD for 180 assayed samples representing paired G0 (original acquisition) and G1 (first regeneration) samples from 90 barley accessions.

Variant	SNPs from BCFtools	SNPs from ANGSD
SNP calling		
Total SNPs	122,463	537,776
SNP annotation with VEP (most severe consequences)		
Missense_variant (MV)	36,926	138,248
Proportion of MV in total SNPs	0.302	0.257
Synonymous_variant (SV)	27,222	98,213
Proportion of SV in total SNPs	0.222	0.183
Splice_acceptor_variant	100	1,988
Splice_donor_variant	131	1,434
Stop_gained	2,646	10,341
Stop_lost	173	525
Start_lost	98	729
Splice_region_variant	48	6,347
Stop_retained_variant	127	126
Coding_sequence_variant	2	4
5_prime_UTR_variant	2,969	28,585
3_prime_UTR_variant	34,700	94,573
Non_coding_transcript_exon_variant	4,975	18,024
Intron_variant	756	33,494
Upstream_gene_variant	1,533	16,211
Downstream_gene_variant	2,347	22,817
Intergenic_variant	6,869	66,117
Loss-of-function (LOF) variant^a^		
Total count	3,323	21,490
Proportion	0.027	0.040
SIFT analysis with CT^b^		
SIFT-deleterious SNPs (SDS)	14,997	49,976
Proportion of SDS in total SNPs	0.122	0.093
Deleterious SNPs by SIFT+RS		
SDS+RS-filtered SNPs (RSD)	2,094	7,508
Proportion of RSD in total SNPs	0.0171	0.0140
Shared RSD between 2 SNP calls		
SNPs with up to 20% missing values	1,642	1,642
SNPs with up to 10% missing values	750	750
SNPs with up to 5% missing values	325	325
SNPs without missing values	9	9

The SNP data set from BCFtools had up to 20% level of missing data in 180 samples, and the ANGSD data set had no missing values.

^a^LOF variants consist of those from the annotation classes of 3 “Stop_,” 3 “Splice_,” and 1 “Start_lost.”

^b^SIFT-filtered with canonical transcripts (CTs).

### Deleterious mutation

Analyzing SIFT scores for SNPs associated with canonical transcripts revealed 14,997 and 49,976 predicted dSNPs for the BCFtools- and ANGSD-based SNP data sets, respectively, as illustrated in Table 2. Combining SIFT scores with GERP++ RS scores identified much fewer, but more accurately predicted, 2,094 and 7,508 dSNPs than those from the SIFT scoring alone for 2 SNP data sets (Table 2). Extra efforts were made to identify dSNPs that were shared between the BCFtools- and ANGSD-based SNP data sets. A total of 1,642, 750, 325, and 9 dSNPs that represented the BCFtools-based SNP data set with the missing levels of 20%, 10%, 5%, and 0%, respectively, were found to be shared with the ANGSD-based SNP data set (Table 2). To make the comparative analysis of mutation burden easier, the first 3 pairs of shared BCFtools- and ANGSD-based dSNP data sets were named as m20-b vs m20s-a, m10-b vs m10s-a, and m5-b vs m5s-a, respectively. The shared 9 dSNPs for 0% missing data were too small to be further considered.

Analyzing the chromosomal distributions of dSNPs revealed their wide distributions across 7 barley chromosomes in m20-b with shared dSNPs (Fig. 1c). Further analysis of their frequency distributions showed similar L-shaped allelic frequency distributions with slightly different means of 0.044 and 0.052 for the m20-b vs m20s-a data set, respectively (Fig. 1d and e). These results meant that most dSNPs were infrequent and harbored in an average of 8 to 9 (out of 180) samples or fewer. Further assessment on the frequency distribution of RS values for shared m20-b revealed that these deleterious alleles had a mean RS value of 0.64 in a range of 0.067 to 1.64 (Fig. 1f). These distributions of RS values indicated that these deleterious alleles were predicted to be weakly detrimental. Only 194 (9.3%) and 126 (6%) dSNPs had RS values greater than 0.64 and 1.00, respectively.

**Fig. 2.**
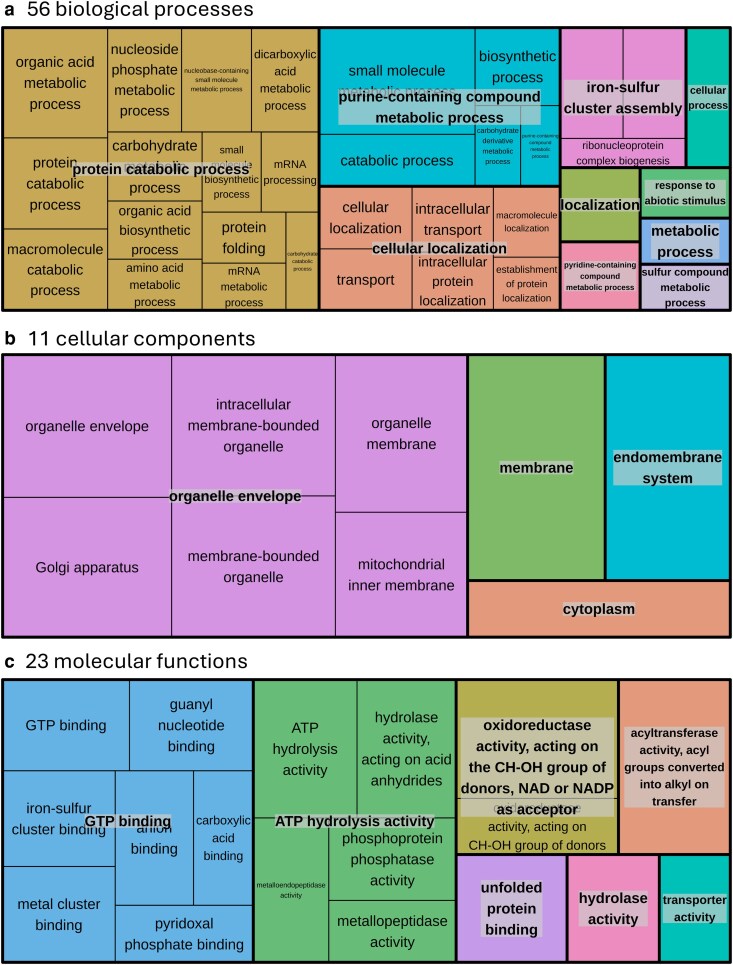
REVIGO gene ontology treemaps showing the cluster representatives of 56 biological processes (a), 11 cellular components (b), and 23 molecular functions (c) associated with 103 significant GO terms that were extracted from 1,025 genes associated with the 1,642 shared dSNPs.

The 1,642 shared dSNPs were found to be associated with 1,025 barley genes across 7 chromosomes. The ontology analyses of the associated genes using agriGO identified 103 significant (*P* < 0.05) GO terms. Analysis of these significant GO terms using REVIGO revealed 56 biological processes (Fig. 2a), 11 cellular components (Fig. 2b), and 23 molecular functions (Fig. 2c) for the associated genes. The major biological processes included protein catabolic process, purine-containing compound metabolic process, cellular localization, iron–sulfur cluster assembly, and response to abiotic stimulus (Fig. 2a). The major cellular components were organelle envelope, membrane, endomembrane system, and cytoplasm (Fig. 2b). The major molecular functions were GTP binding, ATP hydrolysis activity, and oxidoreductase activity acting on the CH-OH group of donors, NAD or NADP as acceptor (Fig. 2c). As expected for stored seeds, these associated genes were mainly involved with protein catabolic process, purine-containing compound metabolic process, response to abiotic stimulus, organelle envelope, membrane, and different molecular bindings.

### Mutation burden change

**Fig. 3.**
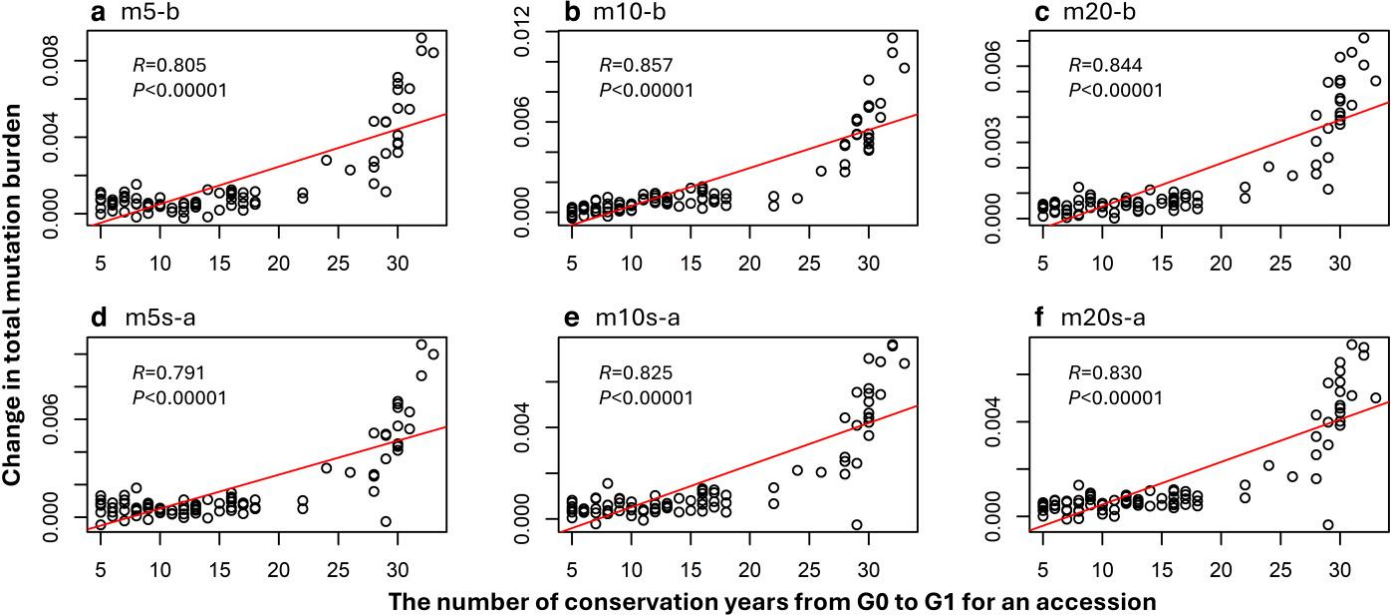
Significant correlations between the estimates of the change in total mutation burden per deleterious locus per conservation year in 1 accession ($DM_{tyi}$) and its conservation years from G0 to G1 in 3 BCFtools-based (a to c) and 3 ANGSD-based (d to f) dSNP data sets.

Linear regression analyses revealed significant correlations between the estimates of the change in total mutation burden per deleterious locus per conservation year in an accession ($DM_{tyi}$) and its conservation years from G0 to G1 in 3 BCFtools-based and 3 ANGSD-based dSNP data sets (Fig. 3). The significance levels obtained were at least 2.0 × 10^−16^, and the estimates of correlation coefficient were positively high, ranging from 0.791 (Fig. 3d) to 0.857 (Fig. 3b). Interestingly, the changes in total mutation burden were largely flat before the conservation years of 25 and started to increase significantly up to the assayed maximum years of 33 (Fig. 3). These results indicated that the number of conservation years from G0 to G1 was a strong indicator for the change in total mutation burden for the assayed barley accessions.

**Fig. 4.**
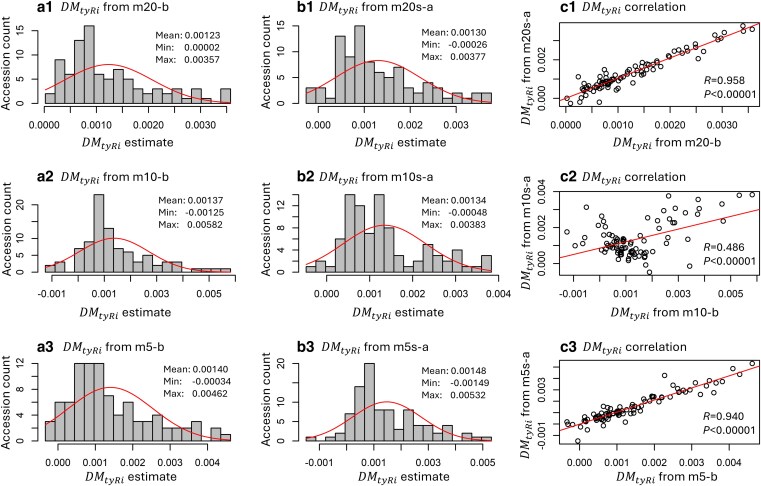
The estimates of the change in total mutation burden per deleterious locus per conservation year over 1 regeneration in an accession ($DM_{tyRi}$) in 3 BCFtools-based dSNP data sets (m20-b, m10-b, and m5-b) (a1, a2, and a3) and their corresponding ANGSD-based dSNP data sets (m20s-a, m10s-a, and m5s-a) (b1, b2, and b3), along with the correlation between BCFtools-based and ANGSD-based $DM_{tyRi}$ estimates (c1, c2, and c3).

The change in total mutation burden per deleterious locus per conservation year between the paired samples of 1 accession with the *Y*_R_ adjustment was estimated as $DM_{tyRi}$ using Equation (3). Accession-wise $DM_{tyRi}$ estimates were obtained for 3 paired dSNP data sets (m20-b vs m20s-a, m10-b vs m10s-a, m5-b vs m5s-a). Analyzing these $DM_{tyRi}$ estimates revealed that these estimates had skewed normal distributions in all 6 dSNP data sets (Fig. 4a and b). Wilcoxon signed-rank tests revealed that accession-wise $DM_{tyRi}$ estimates in each dSNP data set had a median that was significantly (*P* < 0.00001) larger than zero. Regression analyses showed highly significant correlations of these $DM_{tyRi}$ estimates between each pair of dSNP data sets and their estimated correlation coefficients ranged from 0.486 to 0.958 (Fig. 4c).

The comparative statistics of accession-wise $DM_{tyRi}$ estimates were summarized for 3 paired BCFtools-based and ANGSD-based dSNP data sets in Table 3. The average estimates of the accession-wise $DM_{tyRi}$ estimates as $DM_{tyR}$ values were significantly smaller in the BCFtools-based, than the corresponding ANGSD-based, dSNP data sets, except for the pair m10-b vs m10s-a (Table 3). For example, the $DM_{tyR}$ value was 0.00123 and 0.00130 for m20-b and m20s-a, respectively. These positive $DM_{tyR}$ values indicated an accumulation of deleterious variants in conserved barley germplasm after the first regeneration. Interestingly, the $DM_{tyR}$ values ranged from 0.00123 to 0.00140 with a mean of 0.00133 for 3 BCFtools-based dSNP data sets and from 0.00130 to 0.00148 with a mean of 0.00137 for 3 ANGSD-based dSNP data sets, but these $DM_{tyR}$ variations were not significantly (*P* > 0.05) different among 3 BCFtools-based or ANGSD-based dSNP data sets, based on the Kruskal–Wallis tests.

**Table 3.** The comparative statistics of estimating the change in total mutation burden per deleterious locus per conservation year over 1 regeneration in each barley accession ($DM_{tyRi}$) in 6 paired dSNP data sets generated from BCFtools (m20-b, m10-b, and m5-b) and from ANGSD (m20s-a, m10s-a, and m5s-a), along with the results of the Wilcoxon signed-rank tests for differences in $DM_{tyRi}$ between paired dSNP data sets.

Paired dSNP data sets	dSNPs	Missing data (%)	Mean $\left(DM_{tyR}\right)$	Standard deviation	Minimum	Maximum	Rank test (*P*)
m20-b	1642	20	0.00123	0.00084	0.00002	0.00357	0.00550
m20s-a	1642	0	0.00130	0.00092	−0.00027	0.00377	
m10-b	750	10	0.00137	0.00132	−0.00125	0.00582	0.59950
m10s-a	750	0	0.00134	0.00096	−0.00048	0.00383	
m5-b	325	5	0.00140	0.00113	−0.00034	0.00462	0.03214
m5s-a	325	0	0.00148	0.00127	−0.00149	0.00532	

### Allelic changes from G0 to G1 samples

Analyzing the changes of deleterious variants between 90 G0 and 90 G1 samples revealed consistently more deleterious variants predicted in the G1 samples than the G0 samples, as illustrated with the paired dSNP data sets (m20-b vs m20s-a) (Table 4). For example, there were 11,376 and 12,319 dSNPs for the G0 and G1 samples in m20-b, showing a burden change of 0.00319 per deleterious locus without adjusting for variable conservation years among accessions. In contrast, m20s-a had 12,375 and 13,381 dSNPs for the G0 and G1 samples, respectively, having a burden change of 0.00340 per deleterious locus.

**Table 4.** Comparative counting of the deleterious alleles between the paired samples of 90 barley accessions representing the original generation (G0) and the first regeneration (G1) in m20-b and m20s-a data sets sharing 1,642 dSNPs.

Data/sample	Locus/allele	Total	Shared alleles between G0 and G1	New alleles detected in G1	G0 alleles not detected in G1	Estimate of total mutation burden	Estimate of the burden change
m20-b							
G0+G1	Loci	1,642	1,591	17	34		
G0+G1	Alleles	23,695	23,535	65	95		
G0		11,376	11,281		95	0.03849	
		(11,082 + 294)	(10987 + 294)		(95 + 0)		
G1		12,319	12,254	65		0.04168	0.00319
		(11,957 + 362)	(11892 + 362)	(65 + 0)			
m20s-a							
G0+G1	Loci	1,642	1,592	15	35		
G0+G1	Alleles	25,756	25,610	50	96		
G0		12,375	12,279		96	0.04187	
		(12,049 + 326)	(11953 + 326)		(96 + 0)		
G1		13,380	13,331	50		0.04527	0.00340
		(12,985 + 396)	(12,935 + 396)	(50 + 0)			

Two deleterious allelic counts in parentheses represent the numbers of deleterious alleles in heterozygotes and homozygotes in the 90 samples, respectively.

Comparative counting of deleterious variants that were newly detected in the 90 G1 samples and undetected in the 90 G0 samples for these paired dSNP data sets revealed 65 new alleles at 17 loci vs 50 alleles at 15 loci for m20-b vs m20s-a (Table 4). Similar counting of deleterious variants that were present in the 90 G0 samples but undetected in the 90 G1 samples for the paired dSNP data sets revealed 95 undetected alleles at 34 loci vs 96 alleles at 35 loci for m20-b vs m20s-a data sets (Table 4). Clearly, there were fewer newly detected, than undetected, alleles for a smaller share of 1,642 dSNPs. Interestingly, more than 97% of the predicted deleterious variants occurred in heterozygotes in m20-b and m20s-a data sets, and all newly detected and undetected alleles in the G1 samples were present in heterozygotes (Table 4). Clearly, deleterious allelic maintenance and turnover occurred from G0 to G1 samples and an extremely high proportion of the identified deleterious variants harbored in heterozygotes of the assayed samples.

## Discussion

This study represented the first attempt to measure the change in total deleterious mutation burden of barley germplasm conserved under long-term cold storage over 1 regeneration cycle using seed-based RNA-Seq analysis. The analysis identified 1,642 deleterious variants by 2 SNP calling methods (Table 2). These deleterious variants had many genetic features such as wide chromosomal distribution, low allelic frequency, predominantly heterozygous form, association with diverse biological processes, and weak detrimental effect. Based on BCFtools-based SNP calls, the change in total mutation burden per deleterious locus per conservation year was estimated at 0.00123 with a standard deviation of 0.00084 for an accession conserved through the first regeneration (Table 3). More deleterious variants were found in G1 than in G0 samples; variants unique to each sample type were also observed; and a high proportion of variants occurred in heterozygotes (Table 4). These findings not only demonstrate the technical and practical feasibility of measuring mutation accumulation in conserved barley germplasm but also are valuable for enhancing long-term plant germplasm conservation.

The estimated changes in total mutation burden of conserved germplasm are novel and significant, as such estimations have never been made, and they differ in biological meaning and scale from the genome-wide mutation rates commonly estimated for human and other species (eg see The 1000 Genome Project 2011; Sharp et al. 2018). The estimates of the averaged change in total mutation burden per deleterious locus per conservation year were significantly larger than zero. This finding alone has provided the first genomic evidence on the accumulation of deleterious variants in conserved barley germplasm after the first regeneration. Interestingly, the predicted deleterious variants also displayed several informative genetic features, including their presence in only 8 or fewer samples and the occurrence of 97% variants in heterozygotes, their associations with diverse biological processes, and the prediction of weakly deleterious effects. These features are consistent with previous findings of accumulating heterozygous mutation burden in conserved soybean germplasm after up to 10 regenerations (Fu and Horbach 2025) and in other plant genomes (Zhu et al. 2022). Together, these findings confirm that the accumulation of heterozygous deleterious mutations is the major mode of mutation accumulation in conserved germplasm. Based on the genetic theories on deleterious mutations (Charlesworth et al. 1990), they also imply the technical difficulty of eliminating these genome-wide, weakly detrimental variants from conserved germplasm (Fu 1999), thereby increasing the genetic cost associated with maintaining conserved germplasm.

The most significant finding was the estimated extent of deleterious variant accumulation in an accession conserved over 1 regeneration: 0.00123 per deleterious locus per conservation year with a standard deviation of 0.00084. Based on such an extent of mutation burden change with 1,642 loci and averaged 16 yr of conservation for the first regeneration, a barley accession is predicted to accumulate 28 to 37 deleterious variants with a mean of 32 (=0.00123 × 1,642 × 16) over the first regeneration. This predicted range of accumulated deleterious variants was lower than the observed counts of new deleterious variants identified in G1 samples (65 in m20-b and 50 in m20s-a), which did not account for the variable conservation years among the assayed accessions (Table 3). As the estimate of the burden change ($DM_{tyR}$) was scaled to a deleterious locus and a conservation year, it is possible for a genebank manager to make a projection of total mutation burden in germplasm conserved over a period. For example, if the number of averaged conservation years for the first regeneration is 32, not 16 as in this study, there would be 64 deleterious variants projected to be accumulated in the conserved barley germplasm. To make the projection more accurate and useful, however, more measurements of mutation burden change with variable years of conservation are needed to establish a baseline data set of deleterious mutation accumulation for a germplasm collection.

Two methods of SNP calling based on BCFtools and ANGSD were applied with the goal to identify their shared dSNPs and to compare the differences in the estimates of mutation burden change. The use of the shared dSNPs acquired from the BCFtools-based and the ANGSD-based dSNP data sets should improve the accuracy of identifying dSNPs with the SIFT-RS combination alone. The 1,642 shared dSNPs in this study were smaller than the 3,855 dSNPs identified by Kono et al. (2019) from exome capture resequencing of 21 barley lines, but larger than the 1,148 dSNPs identified by Fu et al. (2023) from 70 barley samples and the average of 1,000 deleterious variants per barley accession identified by Kono et al. (2016). As expected, the 1,642 dSNPs were widely spread over 7 chromosomes and associated with 1,025 expressed genes conditioning diverse biological processes, cellular components, and molecular functions. These GO results also revealed a genetic link of some deleterious variants conditioning the response to abiotic stress (probably the cold condition) (Fig. 2a).

A comparison of the estimated changes in total mutation burden per deleterious locus per conservation year between 3 paired BCFtools-based and ANGSD-based dSNP data sets revealed some significantly higher ANGSD-based estimates than BCFtools-based estimates, although the differences were relatively small (Table 3). These higher ANGSD-based estimates are consistent with the larger total number of deleterious alleles in m20s-a (25,756) compared with m20-b (23,695), and with the greater G0 to G1 change in deleterious alleles for m20s-a (1,006) than for m20-b (943) (Table 4). It is worth noting, however, that some allelic changes from G0 to G1 may be false positives caused by sampling effects, as the G0–G1 samples originated from different initial plants and only one pair was sampled for each heterogeneous accession. Also, the higher estimates may reflect mapping biases introduced by allele-frequency optimization procedures designed in ANGSD to genotype low-frequency alleles (Günther et al. 2025), which can enable the detection of rare deleterious alleles that BCFtools-based SNP calling would otherwise classify as missing. In contrast, the lower BCFtools-based estimates were conservative because they were based on the effective deleterious loci defined in Equation (1), which excluded loci with missing data rather than using all 1,642 loci. For example, for a sample with a total number of deleterious variants (for both heterozygotes and homozygotes) counted as 1,500 effective loci and with 142 missing data loci, the total mutation burden per deleterious locus is calculated with the total variant count divided by 3,000 (=1,500 loci × 2), rather than 3,284 (=1,642 loci × 2). Thus, missing data were expected to have less impact on the estimations of the burden change, as supported with nonsignificant differences in $DM_{tyR}$ among m20-b, m10-b, and m5-b data sets. This feature alone tends to favor the choice of BCFtools-based SNP calling for mutation burden analysis, in spite of the strong correlation (*R* = 0.958) between the estimates of the burden changes from m20-b and m20s-a data sets (Fig. 4c).

Germplasm conservation in a genebank is unique with the regular (although not necessarily mandated) maintenance of multiple samples of an accession after germplasm regenerations. This unique feature provides the opportunity to measure the changes of deleterious variants in conserved germplasm. As illustrated in this study, seed-based RNA-Seq analysis of the paired samples of 90 barley accessions representing the germplasm before and after 1 regeneration cycle can allow for estimating mutation burden changes over 1 regeneration cycle (Table 3). However, several issues are worth mentioning for future improvements. First, our study did not consider the variabilities in the number of accessions assayed and in mutation burden within accessions (Fu 2024b). These variabilities could affect the estimation accuracy and/or precision, as each barley accession was genetically heterogenous (due to outcrossing or sample contamination) and represented only by a paired G0–G1 sample that originated from different initial plants. Such a sample pair cannot determine whether an observed allelic turnover was resulted from *de novo* mutation, standing variation, or simply a sampling effect. Thus, future informative analysis should utilize a minimum of 30 accessions, each containing 15 to 30 G0–G1 sample pairs. Second, the variabilities in years of conservation (*Y*_0_, *Y*_1_, and *Y*_R_) for a conserved accession could also affect the estimation of mutation burden change if these conservation years are not linearly correlated with deleterious allelic changes (Fig. 3). More empirical and theoretical research is needed to analyze their impacts of nonlinear correlation on the estimation accuracy. Third, the GERP++ RS scores generated based on reference genomes of 12 plant species (Fu et al. 2023) for 1,642 deleterious variants ranged from 0.07 to 1.64 with a mean of 0.64, suggesting that some predicted deleterious variants, even based on 2 methods of SNP calling, may be false positives (Kistler et al. 2018; Wu et al. 2023). Fourth, representation bias in seed-based RNA-Seq analysis also existed (eg see Shi et al. 2021), even with the sequence alignments by a splice-aware aligner Hisat2 (Kim et al. 2019), and false positives may have also occurred for some predicted deleterious variants.

The reported findings are valuable for enhancing long-term plant germplasm conservation in a genebank. First, the approach developed here for measuring mutation burden changes is technically achievable and practically feasible for its applications to seed germplasm collections of different species conserved in genebanks. There are RNA extraction protocols available for seeds of many plant species (see eg Tetreault et al. 2025), genome sequences for more than 1,800 plant species (Schwacke et al. 2025), and many advanced genomic tools to identify deleterious variants. In this study, the RNA-Seq analysis of 180 barley samples had an experimental cost in 2022 of CAD 18,000 (including RNA extraction kit, library preparation kit, and sequencing), and the whole mutation screening can be completed within a few months from seed RNA extraction, sequencing, to mutation analysis. Second, more research is needed to acquire baseline information on mutation burden changes in seed germplasm of different species conserved under long-term cold storage, as the extent and mechanism of their mutation accumulation after regenerations may differ. Linking mutation burden changes to storage conditions and/or conservation practices should be prioritized, if possible, to enable the development of specific mitigation strategies. Collaborative research among worldwide genebanks is also needed to expand the scope of measuring mutation burden changes with germplasm collections of different species under variable conservation conditions. Third, with the baseline information on mutation burden changes, genebank managers could project the extent of deleterious mutation accumulation in conserved germplasm under variable years of cold storage. Accordingly, effective genebank managements for long-term conservation can be explored to minimize the genetic changes in conserved germplasm (Fu 2024a). For example, for those germplasm collections with a high level of mutation accumulation, existing cold storage conditions may need to be optimized along with long-term regeneration frequency (Richards et al. 2010; Redden and Partington 2018). For long-term conservation involving cycles of germplasm regeneration, genebank managers may utilize estimated mutation burden changes to extrapolate overall genetic changes in conserved germplasm resulting from mutation accumulation alone, expressed as the projected number of accumulative deleterious variants divided by genome size.

### Conclusion

This seed-based RNA-Seq analysis of 90 barley accessions with paired samples representing germplasm conserved under cold storage since its original acquisition (G0) and since the first regeneration (G1) identified 1,642 deleterious variants. These variants were widely distributed across 7 chromosomes, were present in only 8 or fewer samples, occurred predominantly in heterozygotes, were associated with diverse biological processes, and were largely predicted to be weakly detrimental. Based on BCFtools-based SNP calls, the change in total mutation burden per deleterious locus per conservation year was estimated at 0.00123, with a standard deviation of 0.00084, for an accession conserved through the first regeneration. This estimate suggests that a conserved barley accession accumulated 28 to 37 deleterious variants over the G0 to G1 period. More deleterious variants were found in G1 than in G0 samples; variants unique to each sample type were also observed; and a high proportion of variants occurred in heterozygotes. These findings clearly demonstrate the technical and practical feasibility of measuring mutation accumulation in barley germplasm conserved in a genebank.

## Supplementary Material

jkag151_Supplementary_Data

## Data Availability

The raw RNA-Seq data sets generated for this study were deposited in June 2025 into NCBI's SRA database under the BioProject ID PRJNA1274897. Supplemental material available at G3 online.

## References

[jkag151-B1] Andrews S . 2010. FastQC: a quality control tool for high throughput sequence data. https://www.bioinformatics.babraham.ac.uk/projects/fastqc/.

[jkag151-B2] Ashton T . 1956. Genetical aspects of seed storage. In: Owen EB, editors. The storage of seeds for maintenance of viability. Commonwealth Agricultural Bureaux. p. 34–38.

[jkag151-B3] Bertorelle G et al 2022. Genetic load: genomic estimates and applications in non-model animals. Nat Rev Genet. 23:492–503. 10.1038/s41576-022-00448-x. 35136196

[jkag151-B4] Bolger AM, Lohse M, Usadel B. 2014. Trimmomatic: a flexible trimmer for Illumina sequence data. Bioinformatics. 30:2114–2120. 10.1093/bioinformatics/btu170. 24695404 PMC4103590

[jkag151-B5] Charlesworth B, Charlesworth D, Morgan MT. 1990. Genetic loads and estimates of mutation rates in highly inbred plant populations. Nature. 347:308–382. 10.1038/347380a0.

[jkag151-B6] Charlesworth D, Morgan MT, Charlesworth B. 1993. Mutation accumulation in finite outbreeding and inbreeding populations. Genet Res. 61:39–56. 10.1017/S0016672300031086.

[jkag151-B7] Cooper GM et al 2005. Distribution and intensity of constraint in mammalian genomic sequence. Genome Res. 15:901–913. 10.1101/gr.3577405. 15965027 PMC1172034

[jkag151-B8] Crossa J, Hernandez CM, Bretting P, Eberhart SA, Taba S. 1993. Statistical genetic considerations for maintaining germplasm collections. Theor Appl Genet. 86:673–768. 10.1007/BF00222655. 24193775

[jkag151-B9] Danecek P et al 2011. The variant call format and VCFtools. Bioinformatics. 27:2156–2158. 10.1093/bioinformatics/btr330. 21653522 PMC3137218

[jkag151-B10] Danecek P et al 2021. Twelve years of SAMtools and BCFtools. Gigascience. 10:giab008. 10.1093/gigascience/giab008. 33590861 PMC7931819

[jkag151-B11] Davydov EV et al 2010. Identifying a high fraction of the human genome to be under selective constraint using GERP++. PLoS Comput Biol. 6:. 10.1371/journal.pcbi.1001025. PMC2996323 21152010

[jkag151-B12] Dourado AM, Roberts EH. 1984. Phenotypic mutations induced during storage in barley and pea seeds. Ann Bot. 54:781–790. 10.1093/oxfordjournals.aob.a086850.

[jkag151-B13] Dwivedi SL et al 2023. Evolutionary dynamics and adaptive benefits of deleterious mutations in crop gene pools. Trends Plant Sci. 28:685–697. 10.1016/j.tplants.2023.01.006. 36764870

[jkag151-B14] Engels JMM, Ebert AW. 2024. How can we strengthen the global genetic resources’ conservation and use system? Plants. 13:702. 10.3390/plants13050702. 38475548 PMC10934659

[jkag151-B15] Ewels P, Magnusson M, Lundin S, Käller M. 2016. MultiQC: summarize analysis results for multiple tools and samples in a single report. Bioinformatics. 32:3047–3048. 10.1093/bioinformatics/btw354. 27312411 PMC5039924

[jkag151-B16] FAO . 2010. The second report on the state of the world's plant genetic resources. FAO.

[jkag151-B17] FAO . 2014. Genebank standards for plant genetic resources for food and agriculture, Rev. ed. FAO.

[jkag151-B18] FAO . 2022. Practical guide for the application of the genebank standards for plant genetic resources for food and agriculture: conservation of orthodox seeds in seed genebanks. FAO. 10.4060/cc0021en.

[jkag151-B19] Fu YB . 1999. Patterns of purging deleterious genes of synergistic interactions with different breeding schemes. Theor Appl Genet. 98:337–356. 10.1007/s001220051078.

[jkag151-B20] Fu YB . 2024a. Will a plant germplasm accession conserved in a genebank change genetically over time? Front Plant Sci. 15:1437541. 10.3389/fpls.2024.1437541. 39430894 PMC11487523

[jkag151-B21] Fu YB . 2024b. Variability in predicted deleterious mutations among barley accessions conserved ex situ. Crop Sci. 64:3372–3380. 10.1002/csc2.21325.

[jkag151-B22] Fu YB, Horbach C. 2025. Accumulating heterozygous deleterious mutations in conserved soybean germplasm over successive regenerations. Plants. 14:2429. 10.3390/plants14152429. 40805778 PMC12349509

[jkag151-B23] Fu YB, Peterson GW, Horbach C. 2023. Deleterious and adaptive mutations in plant germplasm conserved ex situ. Mol Biol Evol. 40:msad238. 10.1093/molbev/msad238. 37931158 PMC10724023

[jkag151-B24] Günther T, Goldberg A, Schraiber JG. 2025. Estimating allele frequencies, ancestry proportions and genotype likelihoods in the presence of mapping bias. G3 (Bethesda). 15:jkaf172. 10.1093/g3journal/jkaf172. 40737495 PMC12506655

[jkag151-B25] Hay FR, Whitehouse KJ. 2017. Rethinking the approach to viability monitoring in seed genebanks. Conserv Physiol. 5:cox009. 10.1093/conphys/cox009. 28361000 PMC5356937

[jkag151-B26] Henn BM, Botigué LR, Bustamante CD, Clark AG, Gravel S. 2015. Estimating the mutation load in human genomes. Nat Rev Genet. 16:333–343. 10.1038/nrg3931. 25963372 PMC4959039

[jkag151-B27] Hoffman PD, Leonard JM, Lindberg GE, Bollmann SR, Hays JB. 2004. Rapid accumulation of mutations during seed-to-seed propagation of mismatch-repair-defective *Arabidopsis*. Genes Dev. 18:2676–2685. 10.1101/gad.1217204. 15520284 PMC525547

[jkag151-B28] Kim D, Paggi JM, Park C, Bennett C, Salzberg SL. 2019. Graph-based genome alignment and genotyping with HISAT2 and HISAT-genotype. Nat Biotechnol. 37:907–915. 10.1038/s41587-019-0201-4. 31375807 PMC7605509

[jkag151-B29] Kistler L et al 2018. Multiproxy evidence highlights a complex evolutionary legacy of maize in South America. Science. 362:1309–1313. 10.1126/science.aav0207. 30545889

[jkag151-B30] Kondrashov FA, Kondrashov AS. 2010. Measurements of spontaneous rates of mutations in the recent past and in the near future. Philos Trans R Soc B, Biol Sci. 365:1169–1176. 10.1098/rstb.2009.0286. PMC2871817 20308091

[jkag151-B31] Kono TJY et al 2016. The role of deleterious substitutions in crop genomes. Mol Biol Evol. 33:2307–2317. 10.1093/molbev/msw102. 27301592 PMC4989107

[jkag151-B32] Kono TJY et al 2019. The fate of deleterious variants in a barley genomic prediction population. Genetics. 213:1531–1544. 10.1534/genetics.119.302733. 31653677 PMC6893365

[jkag151-B33] Korneliussen TS, Albrechtsen A, Nielsen R. 2014. ANGSD: analysis of next generation sequencing data. BMC Bioinformatics. 15:356. 10.1186/s12859-014-0356-4. 25420514 PMC4248462

[jkag151-B34] Li H et al 2009. The Sequence Alignment/Map format and SAMtools. Bioinformatics. 25:2078–2079. 10.1093/bioinformatics/btp352. 19505943 PMC2723002

[jkag151-B35] Liu Q, Zhou Y, Morrell PL, Gaut BS. 2017. Deleterious variants in Asian rice and the potential cost of domestication. Mol Biol Evol. 34:908–924. 10.1093/molbev/msw296. 28087781

[jkag151-B36] Lozano R et al 2021. Comparative evolutionary genetics of deleterious load in sorghum and maize. Nat Plants. 7:17–24. 10.1038/s41477-020-00834-5. 33452486

[jkag151-B37] Lu J et al 2006. The accumulation of deleterious mutations in rice genomes: a hypothesis on the cost of domestication. Trends Genet. 22:126–131. 10.1016/j.tig.2006.01.004. 16443304

[jkag151-B38] Mascher M et al 2017. A chromosome conformation capture ordered sequence of the barley genome. Nature. 544:427–433. 10.1038/nature22043. 28447635

[jkag151-B39] McLaren W et al 2016. The Ensembl variant effect predictor. Genome Biol. 17:122. 10.1186/s13059-016-0974-4. 27268795 PMC4893825

[jkag151-B40] Mezmouk S, Ross-Ibarra J. 2014. The pattern and distribution of deleterious mutations in maize. G3 (Bethesda). 4:163–171. 10.1534/g3.113.008870. 24281428 PMC3887532

[jkag151-B41] Moyers BT, Morrell PL, McKay JK. 2018. Genetic costs of domestication and improvement. J Hered. 109:103–116. 10.1093/jhered/esx069. 28992310

[jkag151-B42] Mukai T . 1964. The genetic structure of natural populations of *Drosophila melanogaster*. I. Spontaneous mutation rate of polygenes controlling viability. Genetics. 50:1–19. 10.1093/genetics/50.1.1. 14191352 PMC1210633

[jkag151-B43] Naithani S, Geniza M, Jaiswal P. 2017. Variant effect prediction analysis using resources available at Gramene database. In: van Dijk A, editor. Plant genomics databases: methods and protocols. Humana Press. p. 270–297. 10.1007/978-1-4939-6658-5_17. 27987178

[jkag151-B44] Ng PC, Henikoff S. 2003. SIFT: predicting amino acid changes that affect protein function. Nucleic Acids Res. 31:3812–3814. 10.1093/nar/gkg509. 12824425 PMC168916

[jkag151-B45] Plekhanova E, Nuzhdin SV, Utkin LV, Samsonova MG. 2019. Prediction of deleterious mutations in coding regions of mammals with transfer learning. Evol Appl. 12:18–28. 10.1111/eva.12607. 30622632 PMC6304693

[jkag151-B46] Ramu P et al 2017. Cassava haplotype map highlights fixation of deleterious mutations during clonal propagation. Nat Genet. 49:959–963. 10.1038/ng.3845. 28416819

[jkag151-B47] Razifard H et al 2025. The evolutionary dynamics of genetic mutational load throughout tomato domestication history. Mol Ecol. 34:e70024. 10.1111/mec.70024. 40662302 PMC12717970

[jkag151-B48] R Core Team . 2024. R: a language and environment for statistical computing. R Foundation for Statistical Computing. https://www.R-project.org/.

[jkag151-B49] Redden R, Partington D. 2018. Gene bank scheduling of seed regeneration: interim report on a long term storage study. J Integr Agric. 17:1529–1540. 10.1016/S2095-3119(19)62730-9.

[jkag151-B50] Renaut S, Rieseberg LH. 2015. The accumulation of deleterious mutations as a consequence of domestication and improvement in sunflowers and other Compositae crops. Mol Biol Evol. 32:2273–2283. 10.1093/molbev/msv106. 25939650

[jkag151-B51] Richards CM, Lockwood DR, Volk GM, Walters C. 2010. Modeling demographics and genetic diversity in ex situ collections during seed storage and regeneration. Crop Sci. 50:2440–2447. 10.2135/cropsci2010.04.0236.

[jkag151-B52] Roberts EH . 1978. Mutations during seed storage. Acta Hortic. 83:279–282. 10.17660/ActaHortic.1978.83.36.

[jkag151-B53] Roles AJ, Conner JK. 2008. Fitness effects of mutation accumulation in a natural outbred population of wild radish (*Raphanus raphanistrum*): comparison of field and greenhouse environments. Evolution. 62:1066–1075. 10.1111/j.1558-5646.2008.00354.x. 18298643

[jkag151-B54] Schoen DJ, Brown AHD. 2001. The conservation of wild plant species in seed banks. BioScience. 51:960–966. 10.1641/0006-68(2001)051[0960:TCOWPS]2.0.CO;2.

[jkag151-B55] Schoen DJ, David JL, Bataillou TM. 1998. Deleterious mutation accumulation and the regeneration of genetic resources. Proc Natl Acad Sci U S A. 95:394–399. 10.1073/pnas.95.1.394. 9419386 PMC18235

[jkag151-B56] Schwacke R, Bolger ME, Usadel B. 2025. PubPlant—a continuously updated online resource for sequenced and published plant genomes. Front Plant Sci. 16:1603547. 10.3389/fpls.2025.1603547. 40630734 PMC12235464

[jkag151-B57] Sharp NP, Sandell L, James CG, Otto SP. 2018. The genome-wide rate and spectrum of spontaneous mutations differ between haploid and diploid yeast. Proc Natl Acad Sci U S A. 115:E5046–E5055. 10.1073/pnas.1801040115. 29760081 PMC5984525

[jkag151-B58] Shi H et al 2021. Bias in RNA-seq library preparation: current challenges and solutions. BioMed Res Int. 2021:6647597. 10.1155/2021/6647597. 33987443 PMC8079181

[jkag151-B59] Sun S et al 2023. Unraveling prevalence and effects of deleterious mutations in maize elite lines across decades of modern breeding. Mol Biol Evol. 40:msad170. 10.1093/molbev/msad170. 37494285 PMC10414807

[jkag151-B60] Supek F, Bošnjak M, Škunca N, Šmuc T. 2011. REVIGO summarizes and visualizes long lists of gene ontology terms. PLoS One. 6:e21800. 10.1371/journal.pone.0021800. 21789182 PMC3138752

[jkag151-B61] Tetreault HM et al 2025. Assessing the RNA integrity in dry seeds collected from diverse endangered species native to the USA. Front Plant Sci. 16:1585631. 10.3389/fpls.2025.1585631. 40433160 PMC12106311

[jkag151-B62] The 1000 Genomes Project . 2011. Variation in genome-wide mutation rates within and between human families. Nat Genet. 43:712–714. 10.1038/ng.862. 21666693 PMC3322360

[jkag151-B63] Tian T et al 2017. agriGO v2.0: a GO analysis toolkit for the agricultural community, 2017 update. Nucleic Acids Res. 45:W122–W129. 10.1093/nar/gkx382. 28472432 PMC5793732

[jkag151-B64] Valluru R et al 2019. Deleterious mutation burden and its association with complex traits in sorghum (*Sorghum bicolor*). Genetics. 211:1075–1087. 10.1534/genetics.118.301742. 30622134 PMC6404259

[jkag151-B65] Van der Auwera GA et al 2013. From FastQ data to high confidence variant calls: the genome analysis toolkit best practices pipeline. Curr Protoc Bioinform. 43:11.10.1–11.10.33. 10.1002/0471250953.bi1110s43. PMC4243306 25431634

[jkag151-B66] Vaser R, Adusumalli S, Leng SN, Sikic M, Ng PC. 2015. SIFT missense predictions for genomes. Nat Protoc. 11:1–9. 10.1038/nprot.2015.123. 26633127

[jkag151-B67] Wang Z, Gerstein M, Snyder M. 2009. RNA-seq: a revolutionary tool for transcriptomics. Nat Rev Genet. 10:57–63. 10.1038/nrg2484. 19015660 PMC2949280

[jkag151-B68] Wu Y et al 2023. Phylogenomic discovery of deleterious mutations facilitates hybrid potato breeding. Cell. 186:2313–2328. 10.1016/j.cell.2023.04.008. 37146612

[jkag151-B69] Zhu M, Cheng Y, Wu S, Huang X, Qiu J. 2022. Deleterious mutations are characterized by higher genomic heterozygosity than other genic variants in plant genomes. Genomics. 114:110290. 10.1016/j.ygeno.2022.110290. 35124173

