# Supplementary material for: Measuring the change in deleterious mutation burden of barley germplasm conserved over one regeneration cycle
Source: G3 (Bethesda). 2026 Jun 15;16(8):jkag151. doi: 10.1093/g3journal/jkag151 (PMC13439951; doi:10.1093/g3journal/jkag151)
Supplement: Supplementary file 1 [file jkag151_supplementary_data.pdf]

Table S1. Summary of RNA-Seq reads for 90 assayed barley accessions with the paired samples representing the conserved germplasm since its original acquisition (G0; sample label with accession ending with -0) and since the first completed regeneration cycle (G1; sample label with accession ending with -1).

Sequencing label for assayed sample	Original fastq paired reads	Trimmed fastq paired reads	Trimmed paired reads (%)	Total reads in BAM file	Total mapped reads in BAM	Mapped reads (%)	Sequencing label for assayed sample	Original fastq paired	Trimmed fastq paired reads	Trimmed paired reads (%)	Total reads in BAM file	Total mapped reads in BAM	Mapped reads (%)
5_CN09448-0	6152441	5145600	83.6	11410206	8217788	72.0	6_CN09448-1	7200416	6345819	88.1	14112884	10169689	72.1
7_CN09450-0	17758052	3032136	17.1	6940203	4313421	62.2	8_CN09450-1	14871756	3442675	23.1	7905295	4875657	61.7
9_CN09456-0	6893605	6080104	88.2	13685395	7467295	54.6	10_CN09456-1	10063087	9084628	90.3	20569483	12802432	62.2
11_CN09479-0	18738062	3105086	16.6	7098206	4472514	63.0	12_CN09479-1	14091776	3801549	27.0	8750187	5387254	61.6
13_CN09487-0	6769155	5289493	78.1	11707442	8106486	69.2	14_CN09487-1	8732529	8097802	92.7	18395496	11175227	60.7
15_CN12930-0	3234762	2868210	88.7	6266814	4681022	74.7	16_CN12930-1	5492050	4842921	88.2	10850923	6952850	64.1
21_CN13176-0	5118496	4127631	80.6	9213548	6171756	67.0	22_CN13176-1	6077253	4900850	80.6	10755920	7468393	69.4
23_CN13180-0	8543641	6698143	78.4	14879387	10643479	71.5	24_CN13180-1	5923683	4300046	72.6	9618602	5412004	56.3
27_CN13203-0	5956583	5080153	85.3	11170542	7945246	71.1	28_CN13203-1	13099533	11982847	91.5	26243002	17543651	66.9
29_CN13224-0	8194021	6781828	82.8	15223998	9805651	64.4	30_CN13224-1	12933044	11594056	89.6	25590663	17126874	66.9
31_CN13372-0	9530066	8660062	90.9	19292661	13763661	71.3	32_CN13372-1	7344602	6097278	83.0	13450900	8140521	60.5
39_CN14132-0	6700047	5889397	87.9	13137708	9697543	73.8	40_CN14132-1	8330924	7053847	84.7	15459720	10005444	64.7
43_CN14393-0	7164112	6151687	85.9	13793684	9328553	67.6	44_CN14393-1	4515039	3464200	76.7	7747202	4979981	64.3
45_CN14494-0	14921157	3914788	26.2	8995693	5594397	62.2	46_CN14494-1	14007575	2523634	18.0	5783469	3597291	62.2
47_CN14508-0	7215428	6102212	84.6	13783034	9403187	68.2	48_CN14508-1	7009989	6001752	85.6	13518503	8640353	63.9
49_CN14527-0	19899647	3939484	19.8	9029421	5603862	62.1	50_CN14527-1	13300315	3107310	23.4	7088774	4390047	61.9
55_CN14648-0	6600942	4688820	71.0	10686068	6666703	62.4	56_CN14648-1	10361662	9310668	89.9	20809768	13728343	66.0
57_CN14653-0	7334994	5588580	76.2	12554563	8242046	65.6	58_CN14653-1	11515970	10113956	87.8	22292030	15294006	68.6
59_CN14690-0	9066716	7771816	85.7	17269319	11744734	68.0	60_CN14690-1	9480490	7895156	83.3	17619048	12378688	70.3
65_CN14913-0	5871812	4499221	76.6	10128271	6803502	67.2	66_CN14913-1	15041569	13560098	90.2	30484870	20217670	66.3
67_CN15040-0	6265531	5398282	86.2	12450537	8208080	65.9	68_CN15040-1	12645256	11765295	93.0	26063164	17649650	67.7
69_CN15059-0	13506334	3471856	25.7	8001784	4943094	61.8	70_CN15059-1	14507305	3037199	20.9	6922373	4334472	62.6
71_CN15073-0	6843306	6109373	89.3	14018684	9493365	67.7	72_CN15073-1	11999243	11279331	94.0	24872041	17116030	68.8
73_CN15140-0	6138647	5303663	86.4	12068754	7820393	64.8	74_CN15140-1	8649971	7862066	90.9	17739208	12163626	68.6
75_CN16296-0	8753463	8012497	91.5	18231043	12222234	67.0	76_CN16296-1	16797418	15333530	91.3	34119089	23916269	70.1
77_CN16530-0	8122007	7309352	90.0	16779608	11090457	66.1	78_CN16530-1	9781285	9065670	92.7	20455890	14115647	69.0
79_CN16547-0	8917651	7344117	82.4	16640093	11506553	69.1	80_CN16547-1	16090751	14847343	92.3	33479113	23412779	69.9
81_CN16562-0	10170200	9140580	89.9	20987684	13848429	66.0	82_CN16562-1	12707040	11617169	91.4	25878196	18101696	69.9
83_CN16563-0	11194360	10361405	92.6	23460065	15920542	67.9	84_CN16563-1	3466496	2224145	64.2	4913435	2632295	53.6
85_CN16700-0	11628402	10356580	89.1	22815046	15796756	69.2	86_CN16700-1	12178992	11191304	91.9	24751931	16116105	65.1
87_CN16873-0	11485722	10227157	89.0	23858677	15902447	66.7	88_CN16873-1	10554857	9790372	92.8	22008472	15283333	69.4
91_CN17537-0	5565550	4514312	81.1	10308210	6627342	64.3	92_CN17537-1	7647957	6415959	83.9	14268134	9422906	66.0
93_CN17557-0	10279481	8872705	86.3	19820892	13274847	67.0	94_CN17557-1	13591438	12329790	90.7	27588765	18226531	66.1
95_CN17580-0	8798587	7052377	80.2	15835724	10629094	67.1	96_CN17580-1	6238490	4358097	69.9	9805520	6404775	65.3
99_CN17600-0	7251352	6191189	85.4	13872709	9413662	67.9	100_CN17600-1	11264133	9707654	86.2	21530358	14217997	66.0
101_CN17616-0	9498494	8565055	90.2	19170202	13034298	68.0	102_CN17616-1	8566010	7601179	88.7	16879555	11550070	68.4
103_CN17628-0	11879503	10346762	87.1	23302557	15502935	66.5	104_CN17628-1	9463871	8312123	87.8	18585448	12346780	66.4
105_CN17630-0	10043631	8886860	88.5	20393342	13218529	64.8	106_CN17630-1	9403824	8356195	88.9	18925227	12634066	66.8
107_CN17638-0	9155881	8253773	90.1	18454503	12584599	68.2	108_CN17638-1	8974142	7661933	85.4	17142937	11532576	67.3
109_CN17820-0	11020291	8981040	81.5	19934944	14166037	71.1	110_CN17820-1	8612450	7603169	88.3	17071475	11536545	67.6
111_CN17822-0	8243781	6968941	84.5	15571891	10579673	67.9	112_CN17822-1	5192430	3935708	75.8	8855125	5895210	66.6
115_CN31045-0	10043778	7229076	72.0	16275947	10730165	65.9	116_CN31045-1	16978031	8341290	49.1	18836363	12289297	65.2
117_CN31054-0	16615464	2790260	16.8	6405913	3956713	61.8	118_CN31054-1	13393051	3498949	26.1	8042596	4972322	61.8
119_CN31058-0	13766586	7965694	57.9	17881453	11822287	66.1	120_CN31058-1	13501329	7255943	53.7	16339874	10764858	65.9
125_CN31140-0	10771109	8328592	77.3	18722391	12368196	66.1	126_CN31140-1	14629396	9137266	62.5	20609134	13506659	65.5
127_CN31558-0	12083306	2448427	20.3	5602262	3503976	62.5	128_CN31558-1	11094523	2545325	22.9	5848336	3621675	61.9
129_CN31673-0	20761829	11210752	54.0	25110648	16990627	67.7	130_CN31673-1	13971954	6413458	45.9	14420200	9410401	65.3
131_CN32160-0	16854660	2876672	17.1	6553577	4117849	62.8	132_CN32160-1	13055554	3321069	25.4	7633830	4754478	62.3
133_CN32585-0	18096948	9079982	50.2	20412746	13473702	66.0	134_CN32585-1	12639504	5443643	43.1	12277771	8021296	65.3
137_CN33302-0	14590532	9288077	63.7	20930422	13831899	66.1	138_CN33302-1	10445557	5638078	54.0	12628819	8421970	66.7
139_CN33306-0	22767521	12684680	55.7	28496325	18956403	66.5	140_CN33306-1	15932635	6217991	39.0	14008305	9241786	66.0
145_CN35778-0	13587621	4218728	31.0	9438937	6311027	66.9	146_CN35778-1	17830203	5419704	30.4	12158976	8051496	66.2
147_CN35779-0	18994990	4568949	24.1	10277315	6779557	66.0	148_CN35779-1	14629097	4047437	27.7	9083889	6027853	66.4
149_CN35780-0	15363671	4025804	26.2	9049667	5978553	66.1	150_CN35780-1	11473371	3025598	26.4	6803663	4461336	65.6
153_CN39286-0	19004189	3613065	19.0	8234351	5222092	63.4	154_CN39286-1	16716323	4162111	24.9	9560551	5921984	61.9
155_CN39309-0	8563675	2741915	32.0	6142704	4079039	66.4	156_CN39309-1	18227937	5389938	29.6	12057317	8028271	66.6
157_CN39314-0	13955311	4298947	30.8	9645797	6382818	66.2	158_CN39314-1	15042663	4012208	26.7	8980985	5962762	66.4
163_CN39339-0	13387678	4242384	31.7	9511554	6341600	66.7	164_CN39339-1	14036878	5012041	35.7	11281513	7465474	66.2
165_CN39379-0	16854840	5012923	29.7	11224741	7481847	66.7	166_CN39379-1	12126784	4153499	34.3	9304684	6148454	66.1
171_CN39408-0	14811740	2814437	19.0	6424870	4022330	62.6	172_CN39408-1	11657599	3041192	26.1	6985784	4320322	61.8
173_CN39410-0	14095139	2962408	21.0	6530230	4867282	74.5	174_CN39410-1	10233833	4267624	41.7	9767823	6565597	67.2
175_CN39413-0	15608181	2880221	18.5	6584355	4112763	62.5	176_CN39413-1	13551304	3634011	26.8	8317470	5220011	62.8
177_CN40276-0	13093218	8333309	63.6	18238764	13608969	74.6	178_CN40276-1	9519466	3859673	40.5	8690018	5695516	65.5
179_CN40278-0	11106709	5402184	48.6	12023363	8538002	71.0	180_CN40278-1	10322699	4797998	46.5	10743374	7329113	68.2
183_CN40300-0	14954103	2884764	19.3	6598927	4103751	62.2	184_CN40300-1	14205912	3271978	23.0	7510869	4633861	61.7
187_CN42483-0	9454109	4511525	47.7	10359915	6633906	64.0	188_CN42483-1	8840849	3261312	36.9	7657808	4548391	59.4
189_CN42525-0	13189993	6367764	48.3	13901061	10443311	75.1	190_CN42525-1	13010347	11330146	87.1	24521237	18522330	75.5
191_CN42855-0	7012714	4713547	67.2	10288684	7737682	75.2	192_CN42855-1	8789605	6679275	76.0	15224427	9539797	62.7
193_CN42877-0	10236452	7147557	69.8	15685834	11386193	72.6	194_CN42877-1	10041399	5997572	59.7	13759769	8278606	60.2
195_CN42934-0	11471833	6639654	57.9	14448478	11066330	76.6	196_CN42934-1	9626177	6772127	70.4	15650319	9655701	61.7
197_CN43235-0	10045714	3933420	39.2	8451547	6457100	76.4	198_CN43235-1	6310758	4216409	66.8	9547622	5885715	61.6
201_CN43288-0	15626617	2954234	18.9	6772839	4195925	62.0	202_CN43288-1	15115864	4306282	28.5	9882379	6145524	62.2
203_CN43556-0	8032106	2515298	31.3	5677660	3852826	67.9	204_CN43556-1	10483518	6569381	62.7	14749455	9645756	65.4
205_CN43634-0	8688441	5958821	68.6	13229235	9113168	68.9	206_CN43634-1	9297214	5155371	55.5	11359762	6964447	61.3
207_CN43666-0	7873870	3973298	50.5	8824079	6303739	71.4	208_CN43666-1	9110959	4760385	52.2	10847649	6567556	60.5
211_CN43731-0	16525829	3793615	23.0	8704846	5382361	61.8	212_CN43731-1	11217095	2836270	25.3	6566043	3943817	60.1
215_CN43743-0	14267887	3647844	25.6	8396052	5155727	61.4	216_CN43743-1	15399894	4626611	30.0	10607286	6594186	62.2
217_CN43747-0	13514402	4231654	31.3	9740410	6051531	62.1	218_CN43747-1	12121574	3473668	28.7	8003013	4896427	61.2
219_CN45881-0	12577751	3686498	29.3	8478381	5238834	61.8	220_CN45881-1	13618814	3655423	26.8	8429110	5155362	61.2
221_CN45883-0	12635465	3892074	30.8	8971212	5486256	61.2	222_CN45883-1	15514443	4666027	30.1	10734166	6597810	61.5
223_CN45886-0	13931027	2751115	19.7	6291724	3905397	62.1	224_CN45886-1	16445782	4087093	24.9	9382923	5812046	61.9
225_CN45889-0	16909898	4445041	26.3	10202862	6321341	62.0	226_CN45889-1	15182944	4180780	27.5	9610331	5947135	61.9
227_CN45892-0	14293357	4195976	29.4	9660022	5953449	61.6	228_CN45892-1	14238561	3678640	25.8	8481247	5177210	61.0
229_CN46226-0	13424886	3701372	27.6	8508004	5246268	61.7	230_CN46226-1	17640190	3951581	22.4	9067622	5600598	61.8
231_CN46747-0	10617100	2850540	26.8	6550886	4028877	61.5	232_CN46747-1	20480198	4609252	22.5	10560435	6535706	61.9
233_CN46748-0	16756482	4013920	24.0	9211303	5684291	61.7	234_CN46748-1	11797173	3285875	27.9	7537555	4667220	61.9
235_CN46750-0	13095617	3058462	23.4	7047795	4333404	61.5	236_CN46750-1	13937921	2563610	18.4	5886434	3608485	61.3
237_CN46753-0	16697256	3359572	20.1	7706862	4766560	61.8	238_CN46753-1	12484661	3180133	25.5	7316257	4508173	61.6
239_CN51811-0	12148988	3215438	26.5	7379221	4570351	61.9	240_CN51811-1	10092543	2643218	26.2	6094121	3722857	61.1
241_CN52167-0	11813475	2926152	24.8	6736205	4140518	61.5	242_CN52167-1	10474430	2496947	23.8	5761953	3512913	61.0
